# Efficacy of Topical Brinzolamide Treatment in Posterior Microphthalmos- Related Macular Cystoid Lesions: A Case Series

**DOI:** 10.4274/tjo.galenos.2019.40504

**Published:** 2020-03-05

**Authors:** Ceren Durmaz Engin, Umut Baran Ekinci, Alper Selver, Ali Osman Saatci

**Affiliations:** 1 Karadeniz Ereğli State Hospital, Clinic of Ophthalmology, Zonguldak, Turkey; 2 Celal Bayar University Faculty of Engineering, Department of Electrical and Electronics Engineering, Manisa, Turkey; 3 Dokuz Eylül University Faculty of Engineering, Department of Electrical and Electronics Engineering, İzmir, Turkey; 4 Dokuz Eylül University Faculty of Medicine, Department of Ophthalmology, İzmir, Turkey

**Keywords:** Brinzolamide, macular cystoid lesion, retinoschisis, posterior microphthalmos

## Abstract

The aim of this study was to report the outcome of topical brinzolamide 1% treatment on macular cystoid lesions resembling retinoschisis in 4 patients diagnosed with posterior microphthalmia. The medical records of 4 patients with a clinical diagnosis of posterior microphthalmia who had started topical brinzolamide 1% treatment were reviewed. Visual acuity, central foveal thickness, and cystoid lesion area percentage were used to evaluate treatment response. In the follow-up, there was a decrease in central foveal thicknesses and cystoid lesion area percentages in both eyes of 3 of the patients. However, 1 patient showed increases in both parameters. Visual acuity remained stable in 5 eyes and increased in 3 eyes. Topical brinzolamide treatment may have some positive effects on macular cystoid lesions in selected cases.

## Introduction

Microphthalmos is an ocular developmental disorder characterized by an eye with a total axial length of more than two standard deviations smaller than normal for that age group. Posterior microphthalmos (PM) is a type of microphthalmos which is defined as hyperopia, a short axial length, posterior segment foreshortening and a normal-appearing anterior segment of normal or subnormal dimensions.^1,2^

Retinal folds, macular schisis, cystoid lesions, reduced or absent foveal avascular zone, pseudopapilledema and uveal effusion are prominent posterior segment changes in PM.^2,3,4,5,6^ Optical coherence tomography (OCT) scans of PM patients demonstrate that the neurosensory retina is folded, while the retinal pigment epithelium (RPE) layer and choroid are intact without folds.^7,8^ The growth of neurosensory retina occurs independently of other ocular tissues, while choroid and RPE development are regulated by thickened sclera; therefore, disproportionate growth between the neurosensory retina and surrounding outer tissues causes retinal folding.^9,10^ Macular schisis is thought to be caused by thickened sclera, which causes blockage of the trans-scleral outflow.^11,12^

Carbonic anhydrase inhibitors (CAIs) function by acidifying the subretinal space and increasing fluid transport across the RPE; therefore, CAIs have been used for the treatment of macular schisis in various causes.^13^ In this paper, we present 4 cases of PM and evaluate the efficacy of topical brinzolamide 1% treatment on the cystoid cavities resembling retinoschisis.

## Case Report

A chart review was conducted on all patients in our clinic diagnosed with PM who were either being treated with or had previously been treated with topical brinzolamide 1% (Azopt^TM^; Alcon Inc., Belgium) 3 times a day. Four patients (8 eyes) who had been on the treatment for at least 6 months between December 2016 to May 2018 at Dokuz Eylül University, Ophthalmology Clinic, İzmir were included in the study. This retrospective study adhered to the tenets of the Declaration of Helsinki, and informed consent was obtained from all participants. Diagnosis of PM was based on short axial length along with normal corneal diameter and anterior chamber depth. Short axial length was accepted to be between 12.30 mm and 20.36 mm, depending on age, in accordance with the literature.^14^

All patients had undergone a detailed ophthalmological examination, including visual acuity, cycloplegic refraction, corneal topography (Pentacam; *Oculus*, Wetzlar, Germany), spectral domain optical coherence tomography (SD-OCT)-fluorescein angiography (Spectralis; Heidelberg Engineering Ltd, Heidelberg, Germany), and ocular ultrasound (US) (Nidek Co., Japan). We compared the initial and final visual acuity (VA) with a Snellen chart. OCT images were captured in radial scan mode. Initial and final central foveal thickness (CFT) values of the horizontal section crossing over the same macular region in follow-up OCT scans were determined and compared.

In order to evaluate the drug’s effect on macular cystoid lesions more accurately, we calculated the cystoid lesion area (CLA), which was defined as any intraretinal hyporeflective space that was greater than 3x3 pixels. Smaller areas that were not correctly drawn or accurately defined on OCT scans were not included. The area extending from the vitreous-internal limiting membrane interface to the RPE was defined as total retina area (TRA). These areas were detected by hand and converted to gray-scale images. Calculations were made with MATLAB version R2016b, MathWorks, Inc. In accordance with the literature in which MATLAB software was used for segmentation of cyst in SD-OCT images, CLA percentage was calculated as CLA divided by TRA (CLA/TRA).^15,16^ All boundary determinations were assessed by two graders.

All of the patients were admitted to our clinic with a history of low vision. They were healthy according to their medical records and had unremarkable family histories. Due to the lack of family history and consanguineous marriage, we believed all of the cases were sporadic. The patients were questioned for potential adverse effects of the drug at each visit.

VA was stable in 5 eyes of 3 patients. In 3 eyes of 2 patients, VA was increased at the final visit. However, since the initial and final refractions of these patients were different, the effect of the treatment on the increase in VA could not be evaluated. Demographic information, initial cycloplegic refraction, baseline and final VA findings are shown in Table 1. SD-OCT and A and B Scan US results of the patients are summarized in Table 2.

**Figure 1:**
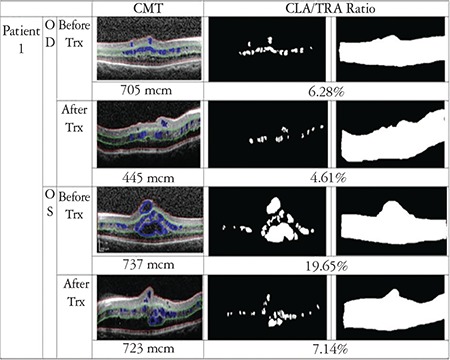
OCT image with manual segmentation lines, cystoid lesion area (CLA) and total retina area (TRA) images of the right and left eyes of patient 1 before and after treatment. Central foveal thickness (CFT) and CLA/TRA values are given with corresponding images

**Figure 2:**
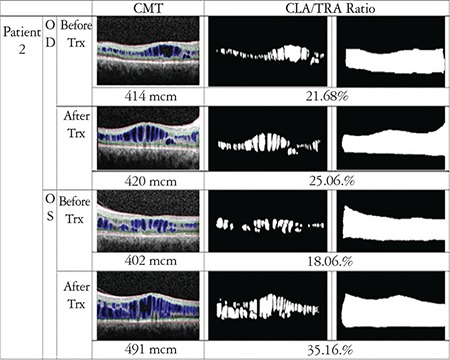
OCT image with manual segmentation lines, cystoid lesion area (CLA) and total retina area (TRA) images of the right and left eyes of patient 2 before and after treatment. Central foveal thickness (CFT) and CLA/TRA values are given with corresponding images

**Figure 3:**
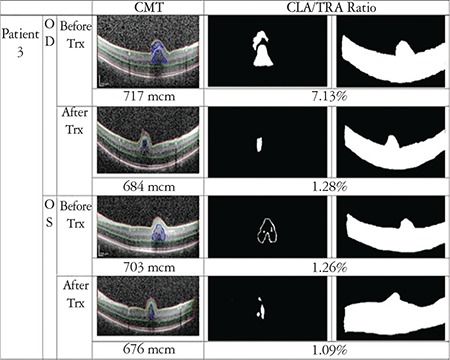
OCT image with manual segmentation lines, cystoid lesion area (CLA) and total retina area (TRA) images of the right and left eyes of patient 3 before and after treatment. Central foveal thickness (CFT) and CLA/TRA values are given with corresponding images

**Figure 4:**
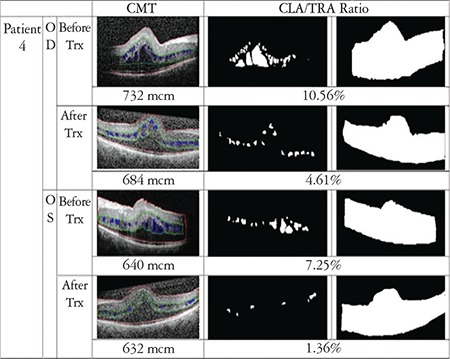
OCT image with manual segmentation lines, cystoid lesion area (CLA) and total retina area (TRA) images of the right and left eyes of patient 4 before and after treatment. Central foveal thickness (CFT) and CLA/TRA values are given with corresponding images

**Figure 5:**
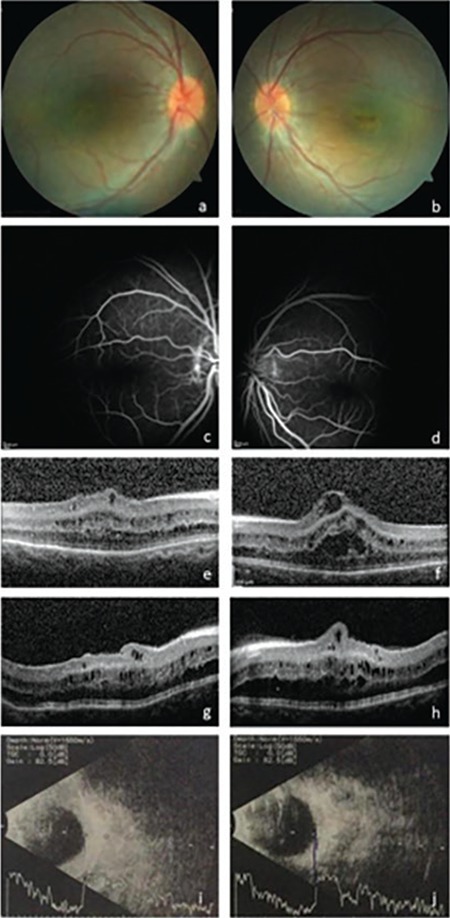
Color fundus photo: mild retinal pigmentary changes and horizontal papillomacular folds bilaterally (a, b). Fundus flourescein angiography: no vascular pathology or cystoid macular edema in both eyes (c, d). Spectral domain optical coherence tomography: retinal folds affecting the neural retina in particular, without involvement of the RPE-choroid band and macular cystoid lesions resembling schisis bilaterally before treatment (e, f) and reduction in cystoid lesion area after treatment (g, h) B-scan ultrasound: diffuse thickening of the choroid and sclera with foreshortening of the globe in both eyes (i, j)

CFT and CLA percentage were found to be decreased in 6 eyes of 3 patients, and increased in both eyes of 1 patient. Results and analyzed images are given in Figure 1, 2, 3 and 4 for patient 1, 2, 3 and 4, respectively. Color fundus photos, late phase of FFA, initial and final OCT scans, and the B-mode US results of Patient 1 are shown in Figure 5.

## Discussion

The carbonic anhydrase (CA) is a group of enzymes found extensively throughout the body. Although CA isoenzymes are diverse, the body’s most common subtype is the intracellular CA isoenzyme II. In the retina, CA II is found in the cytoplasm of red/green cones and inside the Müller cells especially.^17^ However, the RPE appears to contain the membrane-bound isoenzyme IV, which regulates the extracellular pH gradient created via the metabolic activity of cells and also acts as a bicarbonate channel.^18^

CAIs have been proven to increase retinal adhesiveness, enhance the activity of fluid transporter on the RPE cell membrane and change subretinal acidity.^13^ The use of CAIs for retinal diseases is not uncommon. Initial investigations were conducted by Cox et al.^19^ in which 41 patients with cystoid macular edema (CME) of various causes were given oral acetazolamide. The drug was effective in more than half of the patients with uveitis or hereditary outer retinal diseases, but in none with retinal vascular disorders. According to the authors, acetazolamide increased the rate of fluorescein disappearance and fluid transport from vitreous by acting via CA-IV receptors on RPE cells.^19^ A topical CAI for a retinal disease was first used in chronic CME of retinitis pigmentosa (RP) patients and provided partial improvement in visual function.^20^ In 2006, Apushkin et al.^21^ were first to investigate the effect of a topical CAI, in particular dorzolamide 2%, on schisis and cystic macular cavities in X-linked retinoschisis (XLRS). The authors reported more than 7 letters gain in VA within 2 months in nearly half of cases.

Foveoschisis is one of the most common causes of pseudo-CME. Unlike CME, there is no leakage in late phases of FFA in foveoschisis.^22^ The differential diagnosis of foveoschisis includes retinal dystrophies like XLRS, Goldmann-Favre syndrome, enhanced S-cone syndrome, and RP; high myopia; microphthalmia-nanophtalmia; and age-related retinoschisis. The cause of schisis and retinal cysts in hereditary dystrophies is thought to be associated with gene mutations (e.g., in RS1, NR2E3) causing defective protein synthesis. These proteins cause abnormal RPE and PR cell formation and ineffective cellular adhesion, and therefore splitting of the fovea. In PM, however, macular schisis is thought to arise from thickened sclera that causes impermeability of the trans-scleral outflow. Consequent congestion of the retina may result in cystic degeneration by overwhelming Müller cell function, which provides physiologic and architectural support.^11,12^ To the best of our knowledge, our study is first to evaluate the effect of a CAI for PM.

We investigated both the anatomical and functional outcomes of topical brinzolamide treatment. We observed a decrease in CLA percentage in both eyes of 3 patients. However, in both eyes of 1 patient, this ratio was increased. We observed that CLA percentage of this patient was higher than the other patients and cystoid macular degeneration was prominent in his OCT sections. In accordance with the literature, we hypothesized that the drug might have a greater benefit on patients with more preserved retinal anatomy. In patients with healthier RPE and Müller cells, the response to the drug will be better and the cysts can shrink more rapidly.^21^

VA remained stable in 5 eyes and increased in 3 eyes. Reduction in CLA and CFT were not correlated with change in VA except for patient 3. Previous studies in XLRS and RP reported that reduction of CFT was not correlated with improvement in VA.^11,23^

The ages of our cases ranged from 4 to 39 years, and this difference should be considered in terms of drug efficacy and safety. In their study, Khandhadia et al.^24^ examined 4 patients diagnosed with XLRS, 3 of whom were children, and attributed the fact that the only patient who showed continuous clinical improvement with a topical CAI was the adult patient due to his potential high drug compliance. Topical brinzolamide has been reported to be effective when given twice daily in congenital glaucoma cases under 6 years of age and no serious adverse effects have been reported.^25^ Yang et al.^26^ reported that topical brinzolamide used 3 times daily was effective and safe in 4 patients aged 4 to 10 years with the diagnosis of XLRS. However, there is no comparative study between adult and pediatric age group in terms of side effect profile and posology of topical brinzolamide in the literature.

Due to potential requirement for long-term treatment, the use of oral acetazolamide may be limited by potential systemic side effects and a topical drug may be preferred. We used brinzolamide due to its better side effect profile.^27,28^ Karatas et al.^29^ found partial improvement in VA and relative stabilization in CFT in 8 XLRS patients with brinzolamide treatment. In their XLRS case series, Yang et al.^26^ achieved a favorable outcome with topical brinzolamide in the reduction of macular cysts, with no reported adverse drug effects. None of our patients reported any unfavorable side effects.

## Conclusion

Considering the efficacy of topical brinzolamide in the treatment of macular cysts and schisis in XLRS and RP, we have tried to find a cure with brinzolamide in macular cystoid lesions resembling macular schisis of PM in which no treatment is available so far. We observed that topical brinzolamide 1% treatment caused partial reduction in cystoid lesions’ area in PM patients. Prospective studies with longer follow-up period and higher number of patients are needed for evaluating the long-term efficacy. 

## Figures and Tables

**Table 1:**
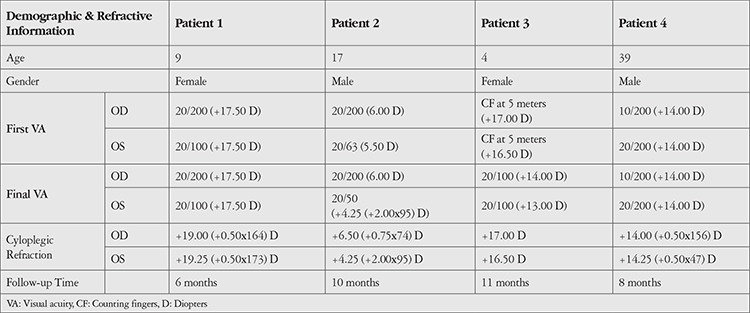
Demographic information, visual acuity, and refractive status of the patients

**Table 2:**
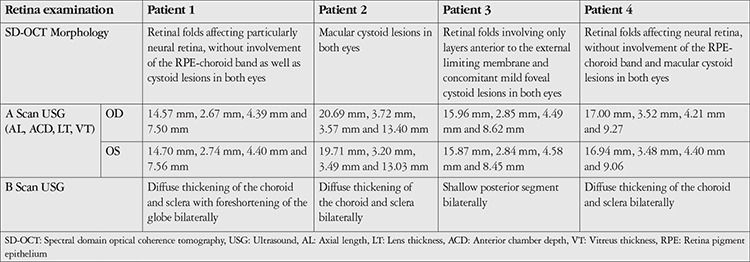
Retina examination results of the patients
